# Metabolic dysfunction‐associated steatotic liver disease, insulin resistance and hepatocellular carcinoma: A deadly triad

**DOI:** 10.1111/eci.70132

**Published:** 2025-10-10

**Authors:** Alfredo Caturano, Enes Erul, Roberto Nilo, Davide Nilo, Vincenzo Russo, Luca Rinaldi, Carlo Acierno, Erman Akkus, Katerina Koudelkova, Federica Cerini, Alessandro Rizzo, Ferdinando Carlo Sasso, Leonilde Bonfrate, Antonio Giordano, Hatime Arzu Yaşar, Caterina Conte

**Affiliations:** ^1^ Department of Human Sciences and Promotion of the Quality of Life San Raffaele Roma University Rome Italy; ^2^ Department of Medical Oncology Ankara University Faculty of Medicine Ankara Turkey; ^3^ Data Collection G‐STeP Research Core Facility, Fondazione Policlinico Universitario A. Gemelli IRCCS Rome Italy; ^4^ Department of Advanced Medical and Surgical Sciences University of Campania Luigi Vanvitelli Naples Italy; ^5^ Department of Biology College of Science and Technology, Sbarro Institute for Cancer Research and Molecular Medicine, Temple University Philadelphia Pennsylvania USA; ^6^ Division of Cardiology, Department of Medical Translational Sciences University of Campania Luigi Vanvitelli Naples Italy; ^7^ Department of Medicine and Health Sciences “Vincenzo Tiberio” University of Molise Campobasso Italy; ^8^ Azienda Ospedaliera Regionale San Carlo Potenza Italy; ^9^ Third Faculty of Medicine Charles University Prague Czech Republic; ^10^ Institute of Metabolic and Cardiovascular Diseases INSERM, Team MetaDiab, University of Toulouse Toulouse France; ^11^ IRCCS MultiMedica Milan Italy; ^12^ Department of Clinical Sciences and Community Health University of Milan Milan Italy; ^13^ S.S.D. C.O.R.O. Bed Management Presa in Carico, TDM, IRCCS Istituto Tumori “Giovanni Paolo II” Bari Italy; ^14^ Department of Medical Biotechnology University of Siena Siena Italy

**Keywords:** cancer progression, hepatocellular carcinoma, insulin resistance, metabolic dysregulation, molecular pathways, tumorigenesis

## Abstract

**Background:**

Metabolic dysfunction‐associated steatotic liver disease (MASLD) has become a leading cause of chronic liver disease worldwide, driven by the increasing prevalence of obesity and insulin resistance (IR). IR, a central feature of the metabolic syndrome, promotes hepatic lipid accumulation, inflammation and mitochondrial dysfunction, fostering the transition from steatosis to advanced liver injury and hepatocellular carcinoma (HCC). This review summarizes current evidence on the molecular mechanisms linking MASLD, IR and HCC, highlighting the role of insulin resistance in liver carcinogenesis and disease progression.

**Methods:**

A comprehensive literature search was conducted to identify experimental, clinical and epidemiological studies addressing the interplay between MASLD, IR and HCC. Key molecular pathways and risk profiles were synthesised and compared across etiologies.

**Results:**

IR contributes to hepatic lipid deposition, oxidative stress and chronic inflammation through activation of PI3K/Akt and mTOR signalling. The coexistence of MASLD and IR enhances pro‐inflammatory and pro‐fibrotic pathways, accelerating the evolution to HCC. Patients with MASLD‐associated HCC exhibit distinct metabolic and molecular characteristics compared with those with viral or alcohol‐related HCC. Novel biomarkers and advanced imaging modalities show promise for identifying high‐risk individuals at earlier disease stages.

**Conclusions:**

Although substantial progress has been made in understanding the MASLD–IR–HCC axis, critical gaps remain regarding genetic, environmental and metabolic determinants. A multidisciplinary approach integrating metabolic, molecular and oncologic research is essential for improving early detection, risk stratification and the development of targeted therapies against metabolic liver cancer.

## Graphical abstract

Metabolic dysfunction‐associated steatotic liver disease (MASLD) is a major global cause of liver disease, driven by obesity and insulin resistance (IR). IR promotes hepatic lipid accumulation, inflammation and mitochondrial dysfunction, accelerating MASLD progression to fibrosis, cirrhosis and hepatocellular carcinoma (HCC). This review examines the molecular interplay between MASLD and IR in hepatocarcinogenesis, highlighting oncogenic pathways (PI3K/Akt, mTOR), pro‐inflammatory and pro‐fibrotic mechanisms and HCC risk profiles. Advances in biomarkers and imaging are discussed alongside strategies for early detection and targeted interventions.

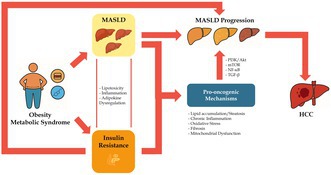

## 1 INTRODUCTION

Metabolic dysfunction‐associated steatotic liver disease (MASLD) represents a significant and growing public health challenge, driven by rising rates of metabolic syndrome worldwide.^1^ MASLD, previously known as nonalcoholic fatty liver disease (NAFLD), encompasses a broad spectrum of liver conditions. The first stage is steatotic liver disease, characterized by ≥5% steatotic hepatocytes. Hepatic steatosis may then progress to metabolic dysfunction‐associated steatohepatitis (MASH), formerly known as nonalcoholic steatohepatitis, which is marked by signs of hepatocellular damage, including cellular ballooning and inflammatory responses, and may or may not involve fibrosis. As the disease advances, it can lead to more severe stages, including cirrhosis, liver failure and ultimately hepatocellular carcinoma (HCC).^2^, ^3^, ^4^ The vast majority of findings on health outcomes in NAFLD cohorts remain valid and transferable under the new MASLD definition.^5^ A central driver in the pathogenesis of MASLD is insulin resistance (IR), a hallmark of metabolic syndrome and a critical factor in hepatic lipid dysregulation and inflammation.^6^ As IR is present in up to 70% of patients with MASLD, it is both a hallmark of the disease and a therapeutic target in MASLD.^7^

The development of HCC within the MASLD spectrum is one of the most severe complications of this disease.^2^ Traditionally associated with cirrhosis secondary to viral hepatitis or alcohol use, HCC increasingly arises in the context of metabolic liver disease, with MASLD‐related HCC cases contributing significantly to the rising global incidence of this cancer.^8^ While viral hepatitis remains the leading cause of HCC worldwide, MASLD is increasingly recognized as a significant etiological factor in regions where viral hepatitis is less prevalent or well‐controlled.^9^ Notably, unlike viral hepatitis‐associated HCC, MASLD‐related HCC often develops even in the absence of cirrhosis, complicating early detection and limiting the effectiveness of traditional surveillance strategies.^10^ The underlying mechanisms linking MASLD to HCC are complex, involving persistent low‐grade inflammation, oxidative stress, dysregulated gut microbiota and activation of tumour‐promoting pathways within the liver.^11^

The global prevalence of MASLD has steadily increased over the past few decades. In recent estimates, the prevalence of MASLD in the general population is approximately 25%–30%, with higher rates in specific regions such as Latin America (44%) and the Middle East (up to 45%).^10^, ^12^ In East Asia, the prevalence is also climbing, largely due to rapid urbanization and lifestyle changes. MASLD disproportionately affects individuals with obesity, type 2 diabetes (T2D) and other metabolic comorbidities, with up to 80%–90% of those with obesity and 50%–70% of those with T2D displaying features of MASLD.^10^, ^13^ The growing prevalence is not limited to adults, as an alarming increase in MASLD among adolescents has been observed, linked to paediatric obesity and IR.^14^

IR is highly prevalent among MASLD patients, with data indicating that as many as 70% of MASLD cases have underlying IR, irrespective of diabetes status.^15^ The increasing global burden of IR is largely attributed to the rise in sedentary lifestyles, high‐calorie diets and obesity.^16^ IR significantly increases the risk of progression from MASLD to MASH and advanced fibrosis, contributing to the severity and complexity of metabolic liver diseases on a global scale.^1^ Concurrently, the incidence of HCC is on the rise, particularly in countries with high prevalence of MASLD and diabetes. MASLD‐related HCC has distinct epidemiological and clinical features. Patients are typically older, with more metabolic comorbidities and more often noncirrhotic than viral/alcohol‐related HCC.^17^ Recent studies estimate that up to 20% of HCC cases in Western countries now arise in the context of MASLD, with some projections suggesting this could exceed 50% in the coming decades due to effective viral hepatitis control.^18^ The incidence of HCC is anticipated to rise further as the global population ages, given that aging exacerbates IR and MASLD progression.^9^

The growing burden of MASLD, IR and HCC underscores the urgency of elucidating the links within this deadly triad. This review aims to explore the complex relationship between MASLD, IR and HCC, offering a comprehensive overview of the epidemiological trends, pathophysiological mechanisms and therapeutic opportunities for these interrelated conditions.

## 2 PATHOPHYSIOLOGY OF MASLD AND THE CENTRAL ROLE OF INSULIN RESISTANCE

### 2.1 General pathophysiology of MASLD

Lipid accumulation within the hepatocytes is the hallmark of MASLD and reflects an imbalance between lipid influx, de novo lipogenesis, fatty acid oxidation and export of very‐low‐density lipoproteins.^19^ This metabolic overload leads to the accumulation of toxic lipid species, such as diacylglycerols, ceramides and lysophosphatidylcholines, which impair cellular homeostasis and trigger hepatocyte injury and apoptosis.^20^ Damaged hepatocytes release damage‐associated molecular patterns, activating innate immune cells, including Kupffer cells and macrophages.^21^ These immune cells, in turn, secrete pro‐inflammatory cytokines, such as tumour necrosis factor‐alpha (TNF‐α), interleukin‐6 (IL‐6) and interleukin‐1β (IL‐1β), generating a pro‐inflammatory microenvironment that perpetuates injury.^22^ Recruited neutrophils and monocytes further amplify this inflammatory cascade.^23^ Activated hepatic stellate cells (HSCs) respond to chronic inflammation by producing extracellular matrix components, initiating fibrosis.^24^ Genetic polymorphisms (*PNPLA3*, *MBOAT7*, *GCKR* and *TM6SF2*) and epigenetic modifications (DNA methylation, histone acetylation) influence susceptibility to advanced disease.^25^, ^26^ Beyond the liver, systemic metabolic dysfunction contributes to disease progression. Reduced adiponectin and elevated leptin promote fibrogenesis, while intestinal dysbiosis facilitates the translocation of microbial products that exacerbate hepatic inflammation via the gut–liver axis.^27^, ^28^

### 2.2 Insulin resistance as the cornerstone of MASLD

IR is present in most patients with MASLD and is considered a central driver of disease progression.^15^ In IR states, peripheral tissues (muscle, adipose) fail to respond adequately to insulin, while paradoxically hepatic insulin signaling continues to promote lipogenesis via persistent activation of SREBP‐1c. This ‘selective IR’ fosters triglyceride accumulation in hepatocytes.^29^, ^30^

**FIGURE 1:**
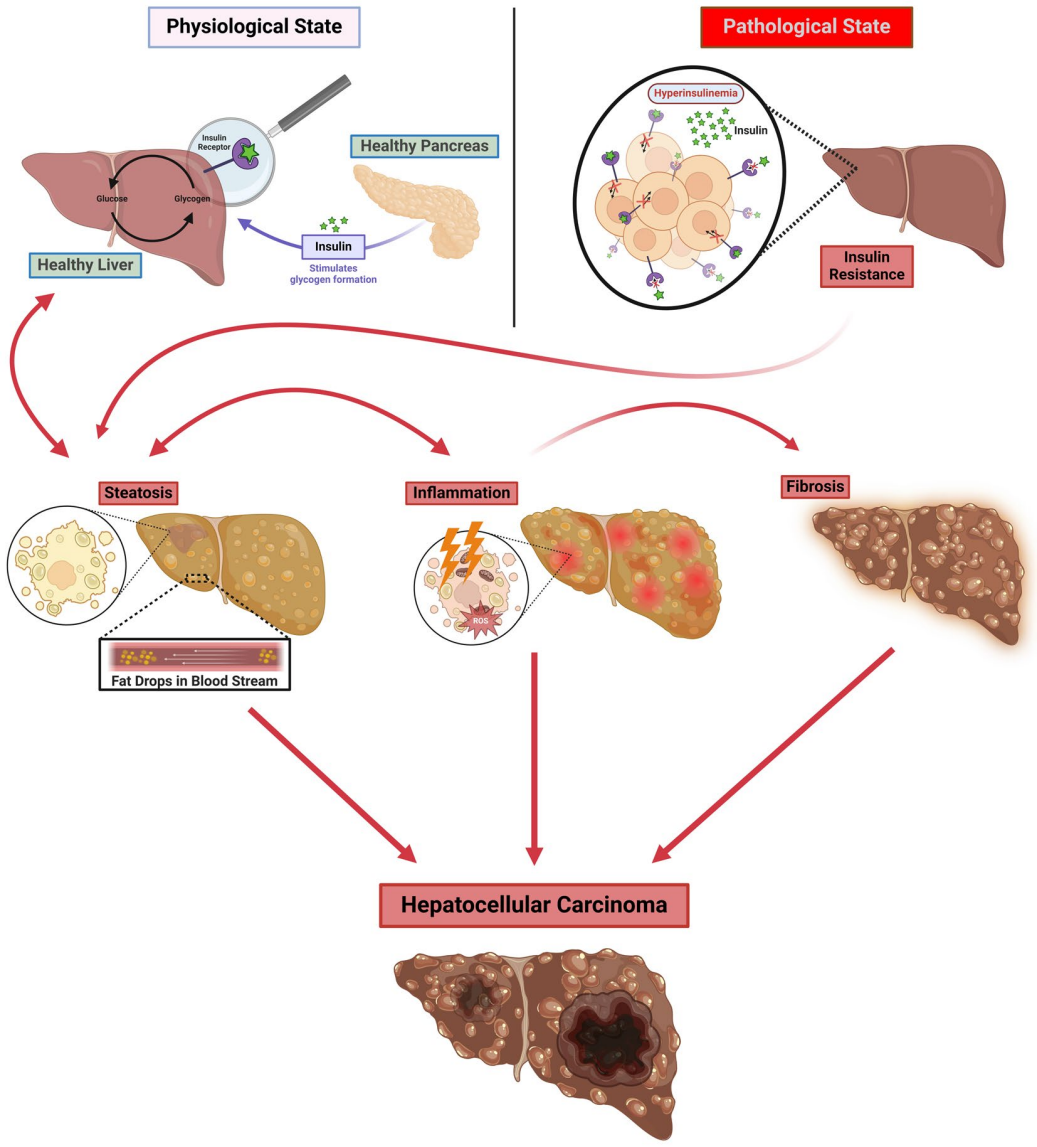
Pathophysiological progression of MASLD: From insulin resistance to hepatocellular carcinoma. The figure illustrates the progression of metabolic dysfunction‐associated steatotic liver disease (MASLD). Under physiological conditions, insulin secreted by the pancreas promotes glycogen synthesis in a healthy liver. However, IR, characterized by hyperinsulinemia and reduced insulin signalling, leads to steatosis with the accumulation of fat droplets in hepatocytes. Persistent steatosis triggers inflammation, reactive oxygen species (ROS) production and oxidative stress, which exacerbate liver damage. Over time, this process contributes to fibrosis, which can progress to hepatocellular carcinoma (HCC). The interconnected stages highlight the impact of IR on liver pathology and the potential for severe outcomes.

In adipose tissue, IR blunts insulin‐mediated suppression of lipolysis, increasing circulating free fatty acids (FFAs).^31^ Excess FFAs are delivered to the liver, overwhelming oxidative and export pathways and enhancing lipotoxicity.^32^ In parallel, inflammatory adipokines (leptin, resistin) and infiltrating macrophages reinforce systemic and hepatic inflammation, further impairing insulin signalling.^33^, ^34^ Thus, IR not only promotes lipid accumulation but also establishes a pro‐inflammatory and pro‐fibrotic milieu that accelerates progression to MASH and fibrosis. Taken together, these IR‐driven mechanisms link systemic metabolic dysfunction with organ‐specific pathomechanisms in MASLD. They illustrate how impaired insulin signalling contributes not only to lipid accumulation but also to inflammation, oxidative damage and fibrosis, reinforcing IR as a central orchestrator of MASLD progression (Figure 1). Moreover, the downstream effects of IR converge on a limited number of interconnected mechanisms that perpetuate liver damage and are reported in Table 1.

**TABLE 1:** Mechanisms Linking Insulin Resistance to MASLD Progression.

Mechanism	Key features	Molecular mediators/Pathways	Clinical impact
Lipotoxicity	Accumulation of toxic lipids beyond hepatocyte storage capacity (ceramides, diacylglycerols, lysophosphatidylcholines)	Activation of PKCε → impaired insulin receptor signalling; ceramide‐induced apoptosis	Hepatocyte death, necroinflammation, transition from steatosis to steatohepatitis
Oxidative stress	ROS generation from mitochondrial β‐oxidation, peroxisomes, CYP2E1 activity	Lipid peroxidation → malondialdehyde (MDA), 4‐HNE; NF‐κB activation	DNA/protein/lipid damage, pro‐fibrogenic signalling, mutagenesis
Mitochondrial dysfunction	Altered morphology, impaired electron transport chain, ATP depletion, defective mitophagy	PGC‐1α dysregulation; decreased oxidative phosphorylation; accumulation of incomplete β‐oxidation products	Energy crisis, ROS amplification, impaired hepatocyte regeneration
Inflammation	Activation of innate immunity by hepatocyte DAMPs and lipids; immune cell recruitment	Kupffer cells, neutrophils, monocytes; cytokines (TNF‐α, IL‐6, IL‐1β); TLR4/MyD88 signalling	Sustained necroinflammation, activation of HSCs, fibrogenesis
Adipokine imbalance	Dysregulated adipose tissue secretome in insulin resistance	↓ Adiponectin (anti‐inflammatory, insulin‐sensitizing); ↑ Leptin, Resistin → JAK/STAT and PI3K pathways	HSC activation, fibrosis progression, systemic inflammation
Gut‐liver axis	Intestinal dysbiosis and increased permeability → translocation of microbial products	LPS → TLR4 activation; altered bile acid signalling (FXR, GPBAR1); ↑ TMAO	Amplification of hepatic inflammation and fibrosis, contribution to carcinogenesis

Abbreviations: 4‐HNE, 4‐hydroxy‐2‐nonenal; ATP, adenosine triphosphate; DAGs, diacylglycerols; DAMPs, damage‐associated molecular patterns; FXR, farnesoid X receptor; GPBAR1, G protein‐coupled bile acid receptor 1 (also known as TGR5); HSCs, hepatic stellate cells; IL‐1β, interleukin‐1 beta; IL‐6, interleukin‐6; IR, insulin resistance; JAK/STAT, Janus kinase/signal transducer and activator of transcription pathway; LPS, lipopolysaccharides; MDA, malondialdehyde; MyD88, myeloid differentiation primary response protein 88; NF‐κB, nuclear factor kappa‐light‐chain‐enhancer of activated B cells; PGC‐1α, peroxisome proliferator‐activated receptor gamma coactivator 1‐alpha; PI3K, phosphatidylinositol 3‐kinase; PKCε, protein kinase C epsilon; RNS, reactive nitrogen species; ROS, reactive oxygen species; SREBP‐1c, sterol regulatory element‐binding protein‐1c; TGF‐β, transforming growth factor beta; TLR4, toll‐like receptor 4; TMAO, trimethylamine‐N‐oxide; TNF‐α, tumour necrosis factor‐alpha.

### 2.3 From steatosis to fibrosis

The transition from simple steatosis to advanced fibrosis reflects the cumulative effect of the mechanisms summarized above.^15^ Lipotoxicity and oxidative stress lead to hepatocyte apoptosis and necrosis, releasing damage‐associated molecular patterns that further activate Kupffer cells and HSCs.^35^, ^36^ Profibrogenic cytokines (TGF‐β, PDGF) sustain myofibroblast activation, resulting in excessive extracellular matrix deposition.^37^ In IR states, systemic inflammation (e.g., circulating TNF‐α and IL‐6 from adipose tissue) contributes to hepatic fibrogenesis.^38^, ^39^ The gut‐liver axis adds another layer: gut dysbiosis contributes to fibrosis in MASLD by increasing intestinal permeability, promoting translocation of microbial products such as lipopolysaccharide, altering microbial metabolite profiles (reduced beneficial short‐chain fatty acids and increased harmful metabolites such as endogenous ethanol, ammonia, trimethylamine‐N‐oxide [TMAO]) and activating hepatic immune and stellate cells.^40^

In summary, IR plays a multifaceted role in the progression of MASLD to fibrosis, driving a pathological cascade that includes lipotoxicity, oxidative stress, inflammation and HSC activation.

## 3 MASLD AND HCC

**FIGURE 2:**
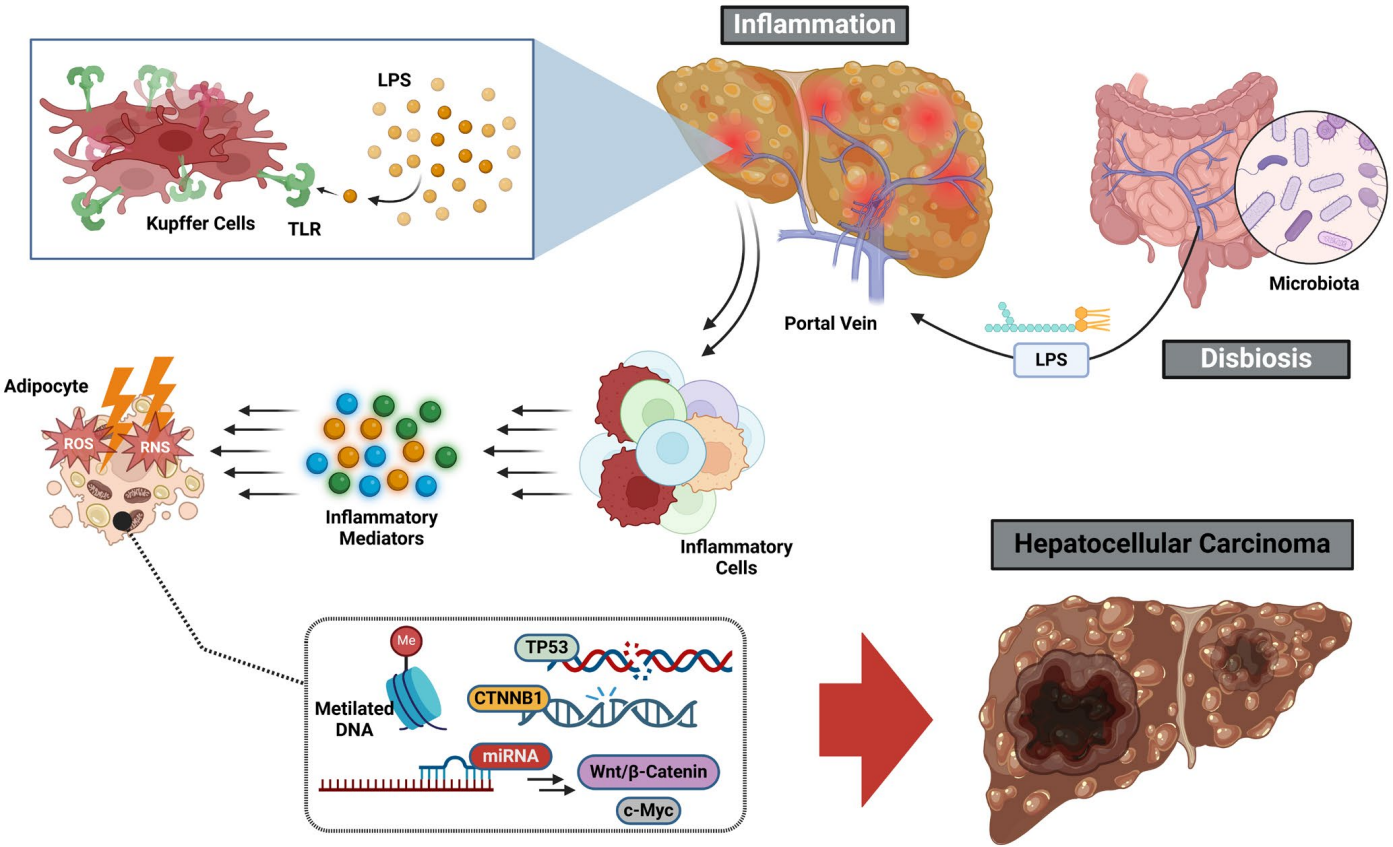
Molecular pathways linking inflammation, oxidative stress and dysbiosis to hepatocellular carcinoma development. The schematic highlights the interconnected mechanisms linking systemic metabolic dysfunction to hepatocellular carcinoma (HCC). Insulin resistance and lipotoxicity promote chronic inflammation, oxidative stress and mitochondrial injury, while gut microbiota dysbiosis amplifies immune activation through lipopolysaccharides (LPS) and altered bile acid signalling. Genetic and epigenetic alterations further destabilize genome integrity, facilitating malignant transformation. Unlike tables summarizing individual determinants, this figure provides a visual map of how these processes converge to create a pro‐tumorigenic microenvironment, bridging molecular insights with clinical outcomes.

MASLD has emerged as a leading cause of HCC, reflecting the combined effects of chronic inflammation, IR and multi‐organ metabolic dysfunction.^41^ The main oncogenic pathways are summarized in Figure 2 and detailed in Table 2.

**TABLE 2:** Molecular and clinical determinants of MASLD‐associated HCC.

Category	Key features	Molecular mediators	Clinical relevance
Chronic inflammation & oxidative stress	Persistent cytokine release, ROS/RNS accumulation	TNF‐α, IL‐6, IL‐1β; NF‐κB, STAT3 activation	Promotes hepatocyte proliferation, DNA damage, mutagenesis
Genetic mutations	High frequency in MASLD‐HCC	TP53, CTNNB1, TERT promoter mutations	Enable immortalization, oncogenic signalling
Epigenetic alterations	DNA methylation, histone modifications, dysregulated microRNAs	↓ miR‐122, ↑ miR‐21; CpG hypermethylation	Loss of tumour suppression, enhanced proliferation/fibrogenesis
Mitochondrial dysfunction	Impaired oxidative phosphorylation, defective mitophagy	Reduced PGC‐1α, ROS amplification	Genomic instability, pro‐tumorigenic environment
Adipokine imbalance	Dysregulated adipose tissue secretome	↑ Leptin, resistin; ↓ Adiponectin	HSC activation, angiogenesis, tumour progression
Gut–liver axis	Dysbiosis, endotoxins, bile acid imbalance	LPS/TLR4, FXR/GPBAR1 dysregulation, ↑ TMAO	Sustained inflammation, carcinogenesis in noncirrhotic livers
Clinical phenotype	MASLD‐HCC often arises without cirrhosis; metabolic syndrome common	Obesity, T2D, hypertension, dyslipidemia	Difficult surveillance; worse prognosis due to comorbidities

Abbreviations: CTNNB1, catenin beta‐1; FXR, farnesoid X receptor; GPBAR1, G protein‐coupled bile acid receptor 1 (also known as TGR5); HSCs, hepatic stellate cells; IL‐1β, interleukin‐1 beta; IL‐6, interleukin‐6; LPS, lipopolysaccharides; miR, microRNA; NF‐κB, nuclear factor kappa‐light‐chain‐enhancer of activated B cells; PGC‐1α, peroxisome proliferator‐activated receptor gamma coactivator 1‐alpha; RNS, reactive nitrogen species; ROS, reactive oxygen species; STAT3, signal transducer and activator of transcription 3; T2D, type 2 diabetes; TERT, telomerase reverse transcriptase; TLR4, toll‐like receptor 4; TMAO, trimethylamine‐N‐oxide; TNF‐α, tumour necrosis factor‐alpha; TP53, tumour protein 53.

### 3.1 Inflammation and oxidative stress as oncogenic drivers

Persistent lipotoxicity and IR create a pro‐inflammatory hepatic environment. Kupffer cells and infiltrating immune cells release cytokines such as TNF‐α, IL‐6 and IL‐1β, activating oncogenic transcription factors including NF‐κB and STAT3.^42^, ^43^ These pathways enhance hepatocyte proliferation, impair apoptosis and allow mutated cells to persist.^42^, ^43^ Simultaneously, reactive oxygen and nitrogen species (ROS/RNS) induce lipid peroxidation and direct DNA damage.^44^ Products such as malondialdehyde (MDA) and 4‐hydroxy‐2‐nonenal (4‐HNE) form mutagenic adducts, contributing to alterations in tumour suppressor genes (*TP53*) and oncogenes (*CTNNB1*).^44^, ^45^, ^46^

### 3.2 Genetic and epigenetic alterations

Genetic mutations represent hallmarks of MASLD‐HCC. TERT promoter mutations are among the earliest and most frequent events, while TP53 and CTNNB1 alterations drive loss of tumor suppression and activation of Wnt/β‐catenin signaling.^45^, ^46^, ^47^ In parallel, epigenetic dysregulation further promotes malignant transformation. Aberrant DNA methylation, characterized by hypermethylation of tumor suppressor gene promoters and hypomethylation of oncogenic loci, leads to profound changes in gene expression.^48^ Altered histone acetylation and methylation similarly modify chromatin accessibility, facilitating transcriptional activation of oncogenic programs.^49^, ^50^ In addition, microRNA profiles are markedly disrupted, with loss of miR‐122, a key tumor suppressor miRNA in the liver, and upregulation of miR‐21, which enhances fibrogenesis and hepatocyte proliferation.^51^, ^52^ Collectively, these genetic and epigenetic alterations cooperate with chronic inflammation and oxidative stress to establish a pro‐oncogenic environment that drives hepatocellular transformation.

### 3.3 Role of the gut–liver axis

Dysbiosis and increased intestinal permeability allow bacterial products, particularly lipopolysaccharides, to translocate to the liver, activating TLR4 signalling in hepatocytes and Kupffer cells.^53^ This amplifies inflammation and fibrosis, fostering a tumour‐promoting microenvironment.^53^ Altered bile acid signalling via FXR and GPBAR1, together with elevated gut‐derived metabolites such as TMAO, contributes to metabolic dysregulation and carcinogenesis.^54^

### 3.4 The interplay between MASLD, insulin resistance and hepatocarcinogenesis

The interplay between MASLD and IR forms a potent axis for HCC development. With the global rise in obesity and T2D, this interaction is increasingly recognized as a critical driver of liver carcinogenesis, particularly in Western countries where it is surpassing viral hepatitis as a dominant risk factor.^1^, ^55^ Both MASLD and IR amplify liver‐specific oncogenic pathways through synergistic pro‐inflammatory and pro‐fibrotic effects, promoting an environment conducive to carcinogenesis^1^, ^55^ (Table 3).

**TABLE 3:** Synergistic Effects of Insulin Resistance in MASLD‐Associated HCC.

Pathway	Effect of MASLD	Effect of insulin resistance	Combined impact on carcinogenesis
Lipotoxicity and lipid overload	Hepatic accumulation of toxic lipids (ceramides, DAGs, lysophosphatidylcholines)	Enhanced adipose tissue lipolysis → ↑ free fatty acids (FFAs) delivered to liver	Exceeds hepatic oxidative capacity, causing hepatocyte apoptosis and chronic injury
Mitochondrial dysfunction and oxidative stress	Impaired β‐oxidation, CYP2E1 activity → ↑ ROS	Hyperinsulinemia drives ROS generation via PI3K/Akt and mTOR	Persistent ROS/RNS → DNA adducts (MDA, 4‐HNE), mutations in TP53/CTNNB1, genomic instability
Inflammation and cytokine signalling	Kupffer cell activation → TNF‐α, IL‐6, IL‐1β secretion	IR sustains NF‐κB/STAT3 activation, reducing apoptosis	Oncogenic transcriptional program: enhanced proliferation, survival of mutated hepatocytes
Fibrogenesis and stromal remodelling	HSC activation via TGF‐β, PDGF → ECM deposition	Adipokine imbalance: ↓ adiponectin, ↑ leptin & resistin → pro‐fibrogenic signalling	Accelerated transition from fibrosis to cirrhosis, creating a pro‐tumour microenvironment
Oncogenic signalling cascades	ROS and inflammation activate Wnt/β‐catenin, c‐Myc pathways	Hyperinsulinemia activates PI3K/Akt/mTOR, MAPK, IGF‐1R signalling	Convergent activation of proliferative and anti‐apoptotic pathways driving HCC
Gut–liver axis	Dysbiosis → LPS translocation, altered bile acids (↓ FXR, ↓ GPBAR1 activity)	IR exacerbates dysbiosis and endotoxemia	Amplified TLR4 signalling, systemic inflammation, carcinogenesis even without cirrhosis
Cellular senescence	Chronic injury → hepatocyte senescence, SASP release	IR accelerates senescence via oxidative stress and nutrient excess	SASP cytokines (IL‐6, IL‐8) sustain inflammation and tumour‐promoting microenvironment
Clinical phenotype	MASLD‐HCC often in noncirrhotic livers	IR associated with T2D, obesity, hypertension	Difficult surveillance; worse prognosis and treatment eligibility

Abbreviations: 4‐HNE, 4‐hydroxy‐2‐nonenal; Akt, protein kinase B; DAGs, diacylglycerols; ECM, extracellular matrix; FFAs, free fatty acids; FXR, farnesoid X receptor; GPBAR1, G protein‐coupled bile acid receptor 1 (TGR5); HSCs, hepatic stellate cells; IGF‐1R, insulin‐like growth factor 1 receptor; IL‐1β, interleukin‐1 beta; IL‐6, interleukin‐6; LPS, lipopolysaccharides; MAPK, mitogen‐activated protein kinase; MDA, malondialdehyde; mTOR, mammalian target of rapamycin; NF‐κB, nuclear factor kappa‐light‐chain‐enhancer of activated B cells; PDGF, platelet‐derived growth factor; PI3K, phosphatidylinositol 3‐kinase; RNS, reactive nitrogen species; ROS, reactive oxygen species; SASP, senescence‐associated secretory phenotype; STAT3, signal transducer and activator of transcription 3; T2D, type 2 diabetes; GF‐β, transforming growth factor beta; TLR4, toll‐like receptor 4; TNF‐α, tumour necrosis factor‐alpha.

### 3.5 Tumour microenvironment and immune dysregulation in MASLD‐HCC

Beyond hepatocyte‐autonomous mechanisms, the tumour microenvironment plays a pivotal role in MASLD‐associated hepatocarcinogenesis.^56^ Chronic low‐grade inflammation and lipotoxic injury reshape the hepatic immune landscape. Cytotoxic CD8+ T cells display features of exhaustion, with impaired effector function and increased expression of inhibitory receptors.^57^ Natural killer cells, critical for anti‐tumour surveillance, exhibit reduced cytolytic activity, while regulatory T cells expand, contributing to immune tolerance. Kupffer cells and infiltrating macrophages undergo phenotypic polarization toward an M2‐skewed profile, secreting IL‐10, TGF‐β and pro‐angiogenic factors. In parallel, senescent hepatocytes release a senescence‐associated secretory phenotype (SASP), characterized by IL‐6, IL‐8 and matrix metalloproteinases, which further promote inflammation and malignant transformation.^57^

### 3.6 Angiogenesis and fibrotic microenvironment

Fibrosis and cirrhosis create a stiff, hypoxic microenvironment that promotes angiogenesis and tumour expansion. Hypoxia‐inducible factor 1‐alpha (HIF‐1α) is stabilized under oxidative stress and metabolic dysfunction, inducing the expression of VEGF and angiopoietins.^58^ Adipokines also contribute: leptin enhances angiogenesis through VEGF upregulation and crosstalk with Notch signalling, while reduced adiponectin removes inhibitory pressure on endothelial proliferation.^59^ The net effect is a pro‐angiogenic microenvironment that sustains tumour growth and metastasis, often making MASLD‐HCC hypervascular and possibly resistant to anti‐angiogenic therapy,^60^ although aetiology‐specific effects are under investigation.

### 3.7 Cellular senescence and regeneration pressure

Repeated cycles of hepatocyte injury and regeneration constitute another carcinogenic driver. Senescent hepatocytes accumulate in MASLD, and their senescence‐associated secretory phenotype secretion promotes local inflammation and stromal remodelling.^61^ Meanwhile, surviving hepatocytes undergo compensatory hyperproliferation to restore parenchymal mass, increasing the likelihood of replication errors and chromosomal instability. This regenerative pressure accelerates clonal selection and expansion of mutated hepatocytes, providing fertile ground for malignant transformation.^61^

### 3.8 Clinical phenotype and prognosis

Clinically, MASLD‐related HCC differs significantly from viral or alcohol‐related HCC. Up to one‐third of cases arise in noncirrhotic livers, limiting the effectiveness of conventional cirrhosis‐based surveillance strategies. These patients often present with larger tumours and more advanced disease at diagnosis.^62^ Moreover, the frequent coexistence of metabolic comorbidities such as T2D, obesity and cardiovascular disease worsens prognosis and narrows therapeutic options.^63^ Cardiovascular risk, for example, may preclude eligibility for liver transplantation, while IR and systemic inflammation reduce tolerance to systemic therapies.^64^, ^65^ Several studies suggest that MASLD‐HCC patients may have inferior overall survival compared with viral‐HCC, though this remains an area of active debate.^66^

### 3.9 Emerging oncogenic pathways

At the molecular level, several interconnected pathways are increasingly recognized as potential drivers of MASLD‐related hepatocarcinogenesis. Among them, the PI3K/Akt/mTOR axis links hyperinsulinemia and nutrient excess to oncogenic signalling, thereby promoting uncontrolled proliferation and resistance to apoptosis.^64^ In addition, Wnt/β‐catenin activation, which is frequently detected in MASLD‐related HCC, acts in concert with JAK/STAT signalling induced by pro‐inflammatory cytokines such as IL‐6 to sustain tumour growth and survival.^64^ The crosstalk between these pathways highlights the complexity of oncogenic networks in metabolic liver disease and underscores the potential for multi‐targeted therapeutic strategies.

### 3.10 Bridging molecular mechanisms to clinical implications

The complex interplay of lipotoxicity, oxidative stress, immune dysregulation and genetic susceptibility in MASLD‐HCC provides crucial insights into patient risk profiles and therapeutic opportunities. These mechanistic pathways explain why MASLD‐related HCC can arise even in noncirrhotic livers and why metabolic comorbidities such as T2D, obesity and dyslipidemia markedly amplify risk.^1^, ^9^, ^10^ Translating these insights into clinical practice highlights the need for refined risk stratification models that go beyond cirrhosis alone, incorporating genetic variants, metabolic traits and systemic inflammation. At the same time, mechanistic overlap with oncogenic signalling pathways underscores potential therapeutic targets, both for metabolic interventions aimed at reducing HCC risk and for systemic treatments once cancer has developed.^43^ This molecular‐to‐clinical continuum is central to the evolving landscape of MASLD‐HCC management and paves the way for precision medicine strategies.

## 4 DETERMINANTS OF RISK IN MASLD‐ASSOCIATED HCC

Risk stratification in MASLD‐associated HCC remains a major challenge, largely because a significant proportion of tumours develop in noncirrhotic livers, thereby escaping traditional cirrhosis‐based models.^1^, ^9^, ^10^ Nonetheless, several risk factors have been consistently identified (Table 4). Demographic variables such as older age are associated with higher incidence rates,^65^ while metabolic comorbidities including T2D, obesity, hypertension and dyslipidemia act synergistically to amplify risk.^67^ Among these, diabetes and poor glycemic control stand out as particularly relevant, approximately doubling the incidence of HCC.^68^ Interestingly, diabetes management drugs appear to modify risk: metformin has been linked to a protective effect, whereas insulin use has been associated with higher cancer incidence.^69^ Obesity represents another important determinant. For every 5 kg/m^2^ increase in BMI, the risk of HCC rises by approximately 1.4‐fold, with central adiposity conferring an even greater effect, nearly tripling the risk compared to leaner individuals.^70^ As expected, advanced fibrosis remains the strongest single predictor of HCC, yet MASLD‐related cancers also emerge in patients without cirrhosis, highlighting the inadequacy of fibrosis alone as a stratification tool.^1^, ^69^ In addition, genetic predispositions contribute to inter‐individual variability. Variants in PNPLA3, TM6SF2, MBOAT7 and GCKR have been shown to increase susceptibility, whereas loss‐of‐function mutations in HSD17B13 appear to exert a protective role.^71^ The integration of such variants into polygenic risk scores has begun to refine stratification, but their translation into routine clinical practice remains limited.

**TABLE 4:** Risk Factors and Stratification Tools for MASLD‐Associated HCC.

Category	Risk factor/Tool	Clinical implications
Demographic	Age ≥ 65 years	Older age = independent risk marker
Metabolic traits	T2D	HbA1c <7% protective; metformin ↓ risk, insulin ↑ risk
	Obesity	Central adiposity more predictive than BMI
	Hypertension & dyslipidemia	Multiplicative impact when combined
	Metabolic syndrome	Most common MASLD‐HCC risk phenotype
Liver disease severity	Advanced fibrosis / cirrhosis	FIB‐4, NFS, elastography valuable for risk prediction
	Noncirrhotic MASLD	Surveillance strategies limited
Genetic factors	PNPLA3 rs738409	Part of PRS
	TM6SF2, MBOAT7, GCKR	Contribute to polygenic models
	HSD17B13 LOF variants	Under therapeutic investigation (ARO‐HSD)
	PRS	Improves stratification when combined with metabolic traits
Pharmacological modifiers	Metformin	May be integrated in risk models
	Statins	Dual benefit (CV + HCC prevention)
Ethnicity/geography	Hispanic ethnicity	Possible genetic‐environmental interaction
	Asian ancestry	Requires regional risk models
Biomarkers/Models	FIB‐4, NFS	Widely used in clinical practice
	Combined clinical + genetic models	Future direction for personalized surveillance

Abbreviations: BMI, body mass index; CV, cardiovascular; FIB‐4, fibrosis‐4 index; GCKR, glucokinase regulatory protein; HbA1c, glycated haemoglobin; HCC, hepatocellular carcinoma; HSD17B13, hydroxysteroid 17‐beta dehydrogenase 13; LOF, loss‐of‐function; MASLD, metabolic dysfunction‐associated steatotic liver disease; MBOAT7, membrane bound O‐acyltransferase domain containing 7; NFS, NAFLD fibrosis score; PNPLA3, patatin‐like phospholipase domain‐containing protein 3; PRS, polygenic risk score; T2D, type 2 diabetes; TM6SF2, transmembrane 6 superfamily member 2.

## 5 THERAPEUTIC STRATEGIES IN MASLD AND MASLD‐ASSOCIATED HCC: CURRENT APPROACHES AND EMERGING PERSPECTIVES

### 5.1 Metabolic and preventive therapies

Lifestyle interventions remain the foundation of MASLD management, with sustained weight loss of 7–10% consistently associated with histological improvement and reduced long‐term liver‐related outcomes. In this context, pharmacological agents traditionally used for diabetes and metabolic disorders have demonstrated ancillary benefits in MASLD.^1^ GLP‐1 receptor agonists promote weight reduction, improve glycemic control and reduce hepatic fat content; semaglutide and liraglutide have shown histological improvement in steatohepatitis in randomized trials. SGLT2 inhibitors improve insulin sensitivity, reduce hepatic steatosis and liver enzymes. Early data suggest possible antifibrotic effects, but confirmatory histology‐based trials are limited. Pioglitazone improves steatohepatitis and fibrosis, while metformin has been associated with reduced HCC incidence, possibly via AMPK activation and inhibition of mTOR signaling. Finally, statins exert pleiotropic effects on lipid metabolism and inflammation and are linked to lower HCC risk in observational studies.^1^, ^72^

While promising, these agents are primarily preventive rather than curative in HCC, and their protective effects must be confirmed in large MASLD‐focused trials.

### 5.2 Treatments under development

Several investigational therapies are being developed for the treatment of MASH/MASLD that could possibly reduce the progression to cirrhosis and HCC. Resmetirom, a selective thyroid hormone receptor‐β agonist, and the GLP‐1 receptor agonist semaglutide have shown efficacy in reducing inflammation and fibrosis in phase III studies^73^ and have received approval by the FDA in the USA for the treatment of MASH with fibrosis, but there is no data on HCC incidence. ARO‐HSD, an RNAi therapeutic silencing HSD17B13, is based on genetic evidence linking loss‐of‐function variants to protection from HCC; early studies show favorable safety and biochemical responses.^71^ Other candidates include FXR agonists (obeticholic acid), FGF21 analogues, and dual incretin agonists, which may indirectly influence carcinogenic risk by reducing fibrosis and systemic metabolic stress.

Although encouraging, these therapies remain investigational, and their impact on HCC incidence is still theoretical.

### 5.3 Systemic therapies for advanced MASLD–HCC

Because MASLD‐HCC frequently arises in noncirrhotic livers and is often detected late, many patients present at stages where systemic therapy is the only viable option. Over the past decade, the treatment landscape of HCC has undergone dramatic change (Table 5).^85^ Tyrosine kinase inhibitors represented the first breakthrough: sorafenib improved overall survival compared with placebo in the SHARP trial and became the first systemic therapy for advanced HCC.^74^ Lenvatinib was later shown to be noninferior to sorafenib, with higher response rates and progression‐free survival.^75^ Both agents have demonstrated efficacy across etiologies, with subgroup analyses showing comparable outcomes in MASLD and non‐MASLD cohorts.^11^

**TABLE 5:** Landmark clinical trials of systemic therapies in advanced HCC.

Treatment line	Study (year)	Agent(s)	Aetiology	Outcome	References
First	SHARP (2007)	Sorafenib vs. placebo	Viral + nonviral; MASLD not studied	OS benefit vs. placebo	^74^
First	REFLECT (2018)	Lenvatinib vs. sorafenib	Broad cohort; MASLD not isolated	Noninferior OS; higher ORR, PFS	^75^
First	IMbrave150 (2020)	Atezolizumab + Bevacizumab vs. sorafenib	MASLD subgroup small, benefit consistent	OS and PFS superiority	^76^
First	HIMALAYA (2022)	Durvalumab + Tremelimumab (STRIDE) vs. sorafenib	Benefit across viral and nonviral	OS superiority	^77^
First	COSMIC‐312 (2022)	Cabozantinib + Atezolizumab vs. sorafenib	No OS benefit in nonviral (incl. MASLD)	PFS gain only, no OS benefit	^78^
First	LEAP‐002 (2023)	Lenvatinib + Pembrolizumab vs. Lenvatinib	Subgroup consistent; MASLD not isolated	Did not meet OS/PFS superiority	^79^
First	CARES‐310 (2023)	Camrelizumab + Rivoceranib vs. sorafenib	Majority HBV (76%); ~15% nonviral (incl. MASLD); benefit consistent	OS superiority (22.1 vs. 15.2 mo, HR .62, *p* < .0001); PFS gain	^80^
First	CheckMate 9DW (2025)	Nivolumab + Ipilimumab vs. lenvatinib/sorafenib	Consistent across HBV, HCV, uninfected (incl. MASLD)	OS superiority (23.7 vs. 20.6 mo, HR .79)	^80^
Second	RESORCE (2017)	Regorafenib vs. placebo	Aetiology‐neutral	OS superiority	^81^
Second	CELESTIAL (2018)	Cabozantinib vs. placebo	Broad etiologies	OS and PFS superiority	^82^
Second	REACH‐2 (2019)	Ramucirumab vs. placebo	Aetiology‐neutral	OS benefit in AFP ≥400 ng/mL	^83^
Second	KEYNOTE‐240/394 (2020–22)	Pembrolizumab vs. placebo	Greater benefit in viral vs. MASLD trend	Mixed: KEYNOTE‐240 negative; KEYNOTE‐394 positive	^84^

Abbreviations: AFP, alpha‐fetoprotein; HBV, hepatitis B virus; HCC, hepatocellular carcinoma; HCV, hepatitis C virus; HR, hazard ratio; ICI, immune checkpoint inhibitor; MASLD, metabolic dysfunction‐associated steatotic liver disease; ORR, objective response rate; OS, overall survival; PD‐1, programmed death‐1; PFS, progression‐free survival; SoC, standard of care; VEGFR2, vascular endothelial growth factor receptor 2.

The most significant progress has come from immunotherapy‐based combinations. Atezolizumab plus bevacizumab (IMbrave150) established a new first‐line standard by significantly improving overall and progression‐free survival compared with sorafenib.^76^ Durvalumab plus tremelimumab (STRIDE regimen, HIMALAYA trial) has also shown superiority over sorafenib and is now an approved alternative.^77^ More recently, nivolumab plus ipilimumab (CheckMate 9DW) and camrelizumab plus rivoceranib (CARES‐310) further expanded first‐line options, with substantial survival benefits compared with sorafenib.^80^ By contrast, cabozantinib plus atezolizumab (COSMIC‐312)^78^ and lenvatinib plus pembrolizumab (LEAP‐002)^79^ failed to meet primary endpoints, though subgroup analyses suggested signals of benefit in certain populations. Second‐line therapies remain important, with regorafenib (RESORCE),^81^ cabozantinib (CELESTIAL),^82^ and ramucirumab (REACH‐2) providing survival benefit, particularly in AFP‐high patients^83^ and pembrolizumab.^84^

### 5.4 Aetiology‐specific considerations

A central debate in the field is whether MASLD‐HCC responds differently to systemic therapy compared with viral‐related HCC. Preclinical studies suggest that immune checkpoint inhibitors may be less effective in MASH‐driven tumours due to immune exhaustion, impaired T cell surveillance and a pro‐inflammatory microenvironment. Indeed, meta‐analyses of clinical trials have raised concerns about reduced immunotherapy benefit in nonviral HCC.^42^ However, post hoc analyses of IMbrave150 did not identify statistically significant differences between etiological subgroups.^11^ In contrast, tyrosine kinase inhibitors (sorafenib, lenvatinib, regorafenib, cabozantinib) and anti‐VEGF therapies (bevacizumab, ramucirumab) appear to exert efficacy largely independent of aetiology, suggesting that their mechanisms of action may bypass immune‐related resistance observed in MASLD.^42^ Combination regimens (e.g., camrelizumab plus rivoceranib, nivolumab plus ipilimumab) have shown survival benefit across etiologies, although dedicated MASLD subgroup analyses remain limited.^60^

This uncertainty underscores the importance of future dedicated studies stratified by aetiology, particularly as MASLD becomes a dominant global cause of HCC. Clarifying whether immune checkpoint therapy is truly less effective in MASLD‐related HCC will be critical for treatment planning and for the design of next‐generation therapeutic strategies.

## 6 KNOWLEDGE GAPS AND FUTURE DIRECTIONS

### 6.1 Current limitations

Despite these advances, several limitations hinder effective risk prediction. Current surveillance is still largely cirrhosis‐based and therefore misses a substantial fraction of MASLD‐related HCC.^86^ Imaging poses another challenge: ultrasound, the most widely used tool, has poor sensitivity in patients with obesity, with up to one in five examinations yielding inadequate results.^87^ Moreover, most patients with MASLD are followed in primary care or diabetes clinics, settings where liver risk assessment is often not prioritized. As a result, many high‐risk individuals remain undetected until late in the disease course.^88^

### 6.2 Emerging tools

Several novel strategies are currently being explored to overcome the limitations of existing surveillance and risk stratification approaches. Among the most promising are noninvasive biomarkers and liquid biopsy techniques. Circulating tumour DNA, extracellular vesicles and RNA signatures have yielded encouraging results in early‐phase studies, suggesting that they could enable earlier detection of HCC in high‐risk patients with MASLD. However, large‐scale validation in MASLD is still lacking.^89^ Another innovation is the use of automated fibrosis scoring. By embedding simple, noninvasive scores such as FIB‐4 or the NAFLD fibrosis score into electronic health records, high‐risk patients can be flagged in real time during routine visits. In cohorts of individuals with T2D, this approach has led to a more than tenfold increase in the detection of advanced fibrosis, illustrating the feasibility of integrating automated alerts into standard care pathways.^90^ In addition, polygenic risk scores are being developed by combining information from multiple genetic variants, including PNPLA3, TM6SF2, MBOAT7 and GCKR. When integrated with clinical and metabolic data, polygenic risk scores may substantially improve the identification of individuals at the highest risk for HCC.^91^ Finally, the advent of artificial intelligence has opened new horizons for hepatology. Deep learning techniques applied to imaging, histology and clinical data have already shown the ability to predict fibrosis severity and estimate HCC risk with impressive accuracy in research settings.^92^ Nevertheless, all these approaches remain in the developmental phase, and prospective validation in large, diverse MASLD populations will be essential before they can be adopted in routine clinical care.

### 6.3 Future directions

Looking forward, several priorities stand out. The first is the development of integrated risk scores that combine clinical, metabolic and genetic factors to generate accurate and individualized predictions. The second is the design of aetiology‐specific surveillance strategies, recognizing that MASLD‐related HCC often arises without cirrhosis, unlike viral hepatitis–driven cancer. Third, there is a pressing need to improve imaging modalities and validate noninvasive biomarkers capable of overcoming the limitations of ultrasound in obese patients. Fourth, large prospective studies are required to test the utility of artificial intelligence‐driven models and liquid biopsy in real‐world MASLD populations. Fifth, improving the recognition of MASLD and increasing the awareness of its association with HCC among healthcare providers is key to enhancing surveillance strategies. These will need to align with therapeutic implications, particularly given the growing evidence that MASLD‐HCC may respond differently to immune checkpoint inhibitors compared with viral‐related disease. To date, however, no phase III RCT has specifically targeted MASLD‐related HCC. Ongoing studies are testing newer agents and immunotherapy combinations in unselected HCC cohorts (viral and nonviral), and results are still pending. There remains a critical need to design and conduct trials tailored to MASLD‐HCC in order to clarify treatment efficacy and optimize therapeutic strategies in this growing patient population. Ultimately, integrating preventive and therapeutic interventions with precision risk prediction represents the most effective strategy to curb the growing burden of MASLD‐associated HCC. A multidisciplinary approach that bridges molecular mechanisms, clinical risk modelling and targeted treatment will be essential to improve outcomes in this expanding patient population.

## AUTHOR CONTRIBUTIONS

Conceptualization, A.C., E.E.; writing‐original draft preparation, A.C., E.E.; writing‐review and editing, A.C., E.E., R.N., D.N., V.R., L.R., C.A., F.C., A.R., F.C.S., L.B., A.G., C.C., A.Y.; supervision, A.Y. and C.C. All authors have read and agreed to the published version of the manuscript.

## FUNDING INFORMATION

This work was supported by the Italian Ministry of Health, Ricerca Corrente IRCCS MultiMedica.

## CONFLICT OF INTEREST STATEMENT

The authors declare no conflict of interest.

## INFORMED CONSENT STATEMENT

Not applicable.

## Data Availability

No dataset was generated for the publication of this article.
